# Modification of transcriptional factor ACE3 enhances protein production in *Trichoderma reesei* in the absence of cellulase gene inducer

**DOI:** 10.1186/s13068-020-01778-w

**Published:** 2020-08-06

**Authors:** Yun Luo, Mari Valkonen, Raymond E. Jackson, Jonathan M. Palmer, Aditya Bhalla, Igor Nikolaev, Markku Saloheimo, Michael Ward

**Affiliations:** 1 DuPont Industrial Biosciences, Genencor Research Center, 925 Page Mill Road, Palo Alto, CA 94304 USA; 2 VTT Technical Research Centre of Finland Ltd, P.O. Box 1000, FI-02044 VTT Espoo, Finland; 3 DuPont Experimental Station, 200 Powder Mill Road, Wilmington, DE 19803 USA; 4 DuPont Industrial Biosciences, Genencor B.V., Willem Einthovenstraat 4, 2342 BH Oegstgeest, The Netherlands

**Keywords:** ACE3 transcription factor, *Trichoderma reesei*, Inducer-free

## Abstract

**Background:**

*Trichoderma reesei* is one of the best-known cellulolytic organisms, producing large quantities of a complete set of extracellular cellulases and hemicellulases for the degradation of lignocellulosic substances. Hence, *T. reesei* is a biotechnically important host and it is used commercially in enzyme production, of both native and foreign origin. Many strategies for producing enzymes in *T. reesei* rely on the *cbh1* and other cellulase gene promoters for high-level expression and these promoters require induction by sophorose, lactose or other inducers for high productivity during manufacturing.

**Results:**

We described an approach for producing high levels of secreted proteins by overexpression of a transcription factor ACE3 in *T. reesei*. We refined the *ace3* gene structure and identified specific ACE3 variants that enable production of secreted cellulases and hemicellulases on glucose as a sole carbon source (i.e., in the absence of an inducer). These specific ACE3 variants contain a full-length Zn_2_Cys_6_ binuclear cluster domain at the N-terminus and a defined length of truncations at the C-terminus. When expressed at a moderate level in the fungal cells, the ACE3 variants can induce high-level expression of cellulases and hemicellulases on glucose (i.e., in the absence of an inducer), and further improve expression on lactose or glucose/sophorose (i.e., in the presence of an inducer). Finally, we demonstrated that this method is applicable to industrial strains and fermentation conditions, improving protein production both in the absence and in the presence of an inducer.

**Conclusions:**

This study demonstrates that overexpression of ACE3 variants enables a high level of protein production in the absence of an inducer, and boosts protein production in the presence of an inducer. It is an efficient approach to increase protein productivity and to reduce manufacturing costs.

## Background

The filamentous fungus *Trichoderma reesei* (teleomorph *Hypocrea jecorina*) is well-known for its ability to secrete large amounts of cellulolytic enzymes. Industrial isolates of *T. reesei* are derived from a single isolate (QM6a) [1, 2] which after several rounds of mutagenesis and selection resulted in mutant strains with elevated levels of cellulase activity including RUT-C30 [3] and RL-P37 [4]. *T. reesei* is now widely used for production of not only its own cellulolytic enzymes, but also foreign proteins (reviewed by [5, 6]). This fungus has a central role as an enzyme producer in biorefineries producing biofuels and chemicals from lignocellulosic biomass.

*Trichoderma reesei* secretes a mix of (hemi)cellulolytic enzymes, with cellulases CBH1, CBH2, EGL1, and EGL2 being the most abundantly produced. The expression of cellulolytic enzymes is highly dependent on available carbon source. It is repressed by glucose and other easily metabolized carbon sources and strongly increased when inducer is provided into growth medium (for review see [7]). The commonly used inducers are cellulose, cellobiose, lactose and sophorose, with sophorose as the most potent known inducer [8–10]. Under inducing conditions, the transcript levels of the main cellulase genes *cbh1*, *cbh2*, *egl1* and *egl2* increase at least 1000-fold, leading to high protein production [11].

Several transcriptional regulators that control transcription of cellulase and hemicellulase genes have been identified so far. The most extensively studied are CRE1, which mediates carbon catabolite repression [12] and XYR1, the major regulator of xylanases and cellulases [13]. Other characterized factors include the positively acting ACE2 [14], CCAAT binding complex HAP2/3/5 [15], as well as the negatively acting factors ACE1, RCE1, and RXE1 [16–18]. XYR1 has been identified as the main transcriptional activator for the expression of cellulases and hemicellulases as well as many other genes [19]. It recognizes specific binding sites with the consensus core 5′-GGCTAR-3′ within promoter regions of target genes and activates their transcription. Deletion of the *xyr1* gene results in a dramatic decrease of cellulase and hemicellulase production [13]. Another essential transcriptional activator, ACE3, has been identified from transcriptional profiling data of *T. reesei* cultures grown under induction conditions [20]. Overexpression of this gene improved both cellulase and hemicellulase production, whereas *ace3* deletion abolished cellulase production and slightly reduced expression of hemicellulases. Expression of *xyr1* and *ace3* is coordinated, as the transcription of *xyr1* is increased in a strain overexpressing *ace3* and decreased in a strain deleted for *ace3* [20, 21]. The transcription of *ace3* was slightly decreased in a *xyr1* deletion strain [22]. Zhang et al. [21] recently demonstrated that ACE3 binds to the specific motifs 5′-CGGAN(T/A)_3_-3′ which were found in a number of cellulase-related genes including *cbh1, xyr1, ace3,* and *crt1*. The authors also proposed that ACE3 and XYR1 may interact through their corresponding C-termini, forming homodimers and/or a heterodimer complex. Interestingly, an 11-amino acid truncation due to a premature stop codon at the ACE3 C-terminus that arose in strain NG14 during a mutagenesis experiment by Sheir-Ness and Monentencourt [4] has been shown to account for increased cellulase production in strain NG14 and its derivative strains of RUT C-30 and RL-P37 [23]. In addition, there is an atypically large intron near the 5′ end of the *ace3* gene. While introns do not encode protein products, they can be integral to gene expression regulation. Furthermore, some introns play roles in a wide range of gene expression regulatory functions such as non-sense mediated decay and mRNA export (reviewed in [24]).

Production of cellulases and hemicellulases with a low-cost carbon source is desirable in the enzyme industry. Glucose is a preferred carbon source as it is cheap and highly soluble, but it represses protein production due to carbon catabolite repression and lacks an inducing property. Currently, the industry relies on lactose or cellulose for induction during large-scale fermentation or may apply a mix of sugars including glucose/sophorose to be used as feed in order to reduce cost [25]. So far, the main strategy to allow (hemi)cellulase production without inducer has been focused on manipulating the essential transcriptional factor XYR1. A publication by Wang et al. [26] reported that constitutive overexpression of *xyr1* increased cellulase activity in strain RUT-C30 grown on glucose. However, when tested in 14-L fed-batch fermentation with glucose as the sole carbon source, constitutive expression of *xyr1* did not increase cellulase production in an RUT-C30 derived industrial strain [27]. Derntl et al. [28] found a single amino acid change in XYR1 (A824V) resulting in a constitutively active form that strongly deregulated xylanase gene expression and increased the basal level of cellulase gene expression on glucose. A subsequent study by Ellilä et al. [29] utilized a similar mutation, V821F, to obtain a high level of cellulase expression on glucose. Interestingly, these mutations are analogous to the XlnR (V756F) mutation in *Aspergillus niger*, which results in constitutive xylanase expression even under repressing conditions [30]. Derntl et al. [31] constructed a hybrid transcriptional factor by fusing the DNA-binding domain of XYR1 and the transactivation domain of another transcriptional factor YPR1, and showed induced xylanase and cellulase expression on glucose and on glycerol. Despite the fact that these strategies demonstrated various levels of success in increasing protein production without an inducer, the total level of secreted proteins remained lower than the one observed with the parental strain under inducing conditions.

In this work, we refined the *ace3* gene structure and identified two independent transcripts at the *ace3* gene locus. We showed that, when overexpressed at the appropriate level, an ACE3 variant comprising an intact Zn_2_Cys_6_ binuclear cluster domain in combination with a defined truncation at the C-terminus enabled a comparable or higher level production of secreted (hemi)cellulases production on glucose (non-inducing condition), and a further elevated level of production on lactose (inducing condition) when compared to that with the parental strain. The roles of the different N-termini, C-termini, and the large intron were addressed by expressing different variants of the *ace3* gene in *T. reesei* strain RL-P37. Overexpression of the different variants of the *ace3* gene was also tested in combination with deletion of the native *ace3* gene and in combination with deletion or overexpression of *xyr1*. Finally, we evaluated the benefit of *ace3* overexpression in industrial strain fermentation.

## Results

### Identification of the *ace3* gene structure and transcripts

**Fig. 1:**
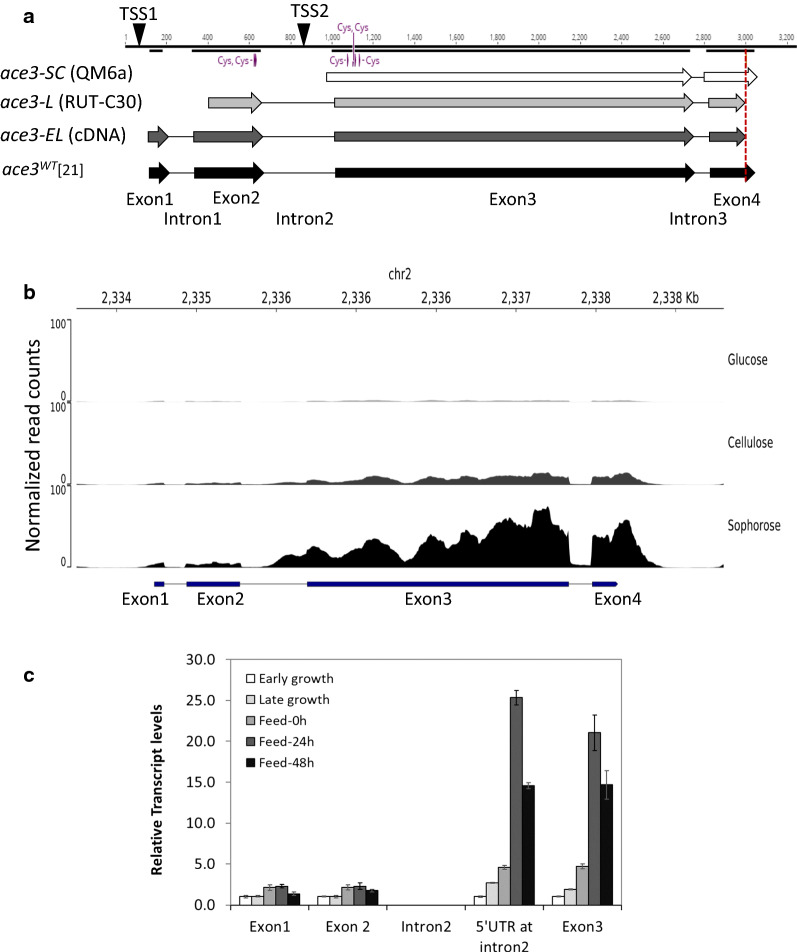
Gene structure and transcripts at the *ace3* locus. **a** Schematic presentation of *ace3* gene annotations at its native locus. Exons are shown as arrows and introns as single lines. In parenthesis are the origins of gene annotations. ▼, Transcriptional start site (TSS); dashed line, non-sense mutation; Cys: cysteine of the Zn_2_Cys_6_ binuclear domain. **b** Transcription levels of *ace3* exons in strain QM9414 grown on different carbon sources for 24 h, as measured by RNA-seq. Normalized read counts are graphed using 100-bp bins per million mapped reads (BPM). **c** RT-qPCR analysis of expression levels of *ace3* exons, Intron 2, and 5′UTR part of Intron 2. The 5′UTR is located within intron 2 and upstream of the *ace3*-*SC* ORF. The transcript expression levels are normalized to the house keeping gene *act1*, and are shown as relative ratios to the level of exon 1 at early growth phase. The mean ± standard deviation was calculated from three independent experiments

The *ace3* gene has two different annotations of the 5′ part in the publicly available genome sequences of strains QM6a and RUT-C30, although the nucleotide sequences are the same (https://genome.jgi.doe.gov/). Further analysis of the genomic DNA sequence and additional cDNA sequence data predicts even a longer *ace3* open reading frame than the two annotations (data not shown). Hereafter, we refer to the predicted proteins derived from the QM6a and RUT-C30 annotations, and the one supported by cDNA cloning as the ACE3-SC, ACE3-L and ACE3-EL variants, respectively (Fig. 1a). In this study, we used strain RL-P37 and its derivative strains, which contain the same 3′ premature stop codon as the strain RUT-C30 (Fig. 1a dashed line). The wild-type ACE3 suggested by Zhang et al. [21] shares the same N-terminus as the ACE3-EL variant but contains an un-truncated C-terminus.

To dissect the transcripts at the *ace3* locus, we performed 5′-RACE (rapid amplification of 5′-cDNA end) to map the *ace3* transcription start sites. RNA samples isolated at 48 h time point from shake flask cultures grown under induced conditions (in NREL medium supplemented with 2.5% glucose/sophorose) were used as templates for amplification in combination with nested reverse primers specific to exon 3 to capture all upstream transcripts. We identified two different transcripts at the *ace3* locus (Additional file 1: Fig S1). One transcript was mapped to initiate at a position ~ 78 bp upstream of the start codon of the *ace3*-*EL* ORF (TSS1, Fig. 1a), and the other was mapped to start within intron 2 at ~ 148 bp upstream of the presumptive start codon of the *ace3*-*SC* ORF (TSS2, Fig. 1a). The presence of the 5′ UTR in these two transcripts suggests that they do not originate from alternative splicing, but from two different transcription start sites.

We examined the abundance of *ace3* transcripts using published RNA-seq data from strain QM9414 fed with glucose, cellulose or glucose/sophorose [32]. As shown in Fig. 1b, the transcripts of four *ace3* exons were low on glucose, moderate on cellulose, and prominent on glucose/sophorose. Surprisingly, mRNA levels corresponding to exons 3 and 4 were increased by ~ 40-fold after sophorose induction, while reads mapped to exons 1 and 2 were merely increased by ~ twofold, suggesting that the short transcript SC was highly induced by sophorose, while the long EL transcript was only moderately induced by sophorose (Fig. 1b).

To verify the expression profiles of the *ace3* specific transcripts in our strain RL-P37, we performed RT-qPCR using RNA isolated from different fermentation stages, including early and late growth phase with glucose, the onset of glucose/sophorose feed (0 h), and 24 h and 48 h after feed start. We quantified the relative abundance of mRNAs corresponding to each exon and found that transcripts from exon 1 and exon 2 were induced ~ twofold by sophorose, whereas the transcripts covering the region of exon 3 and partly intron 2 were increased by 20- to 25-fold (Fig. 1c). Together these results suggest that there are at least two independent transcripts at the *ace3* locus. The SC transcript is transcribed most abundantly upon sophorose induction. It starts within intron 2 and expands to the region of the last two exons 3 and 4. Interestingly, a single XYR1 binding site is located 186 bp upstream of the defined start of transcription, just at the 5′ border of intron 2. Whether this accounts for a sophorose-dependent induction profile of the SC transcript remains unclear. No consensus TATA box of the core promoter was found within intron 2, and no expression of a marker gene was detected when the long intron was fused to the mCherry gene (data not shown). It remains unclear whether the transcript SC gets translated into a functional protein. In contrast, intron 2 contains long pyrimidine stretches which are known for their role in mediating transcription. The observation that transcript SC appeared to be tenfold more abundant than transcript EL is rather unexpected. The EL transcript is transcribed from a promoter located upstream of exon 1 that only weakly responds to sophorose and covers all four exons. It contains two in-frame open reading frames for *ace3*-*EL* and *ace3*-*L* encoded proteins, which both contain an intact Zn_2_Cys_6_ domain. A separate *ace3*-*L* transcript (comprising exon 2, 3 and 4), however, was not detected by the methods used in this study and is likely an artifact from RUT-C30 genome annotation.

### Overexpression of *ace3* enables secreted protein production on glucose

**Fig. 2:**
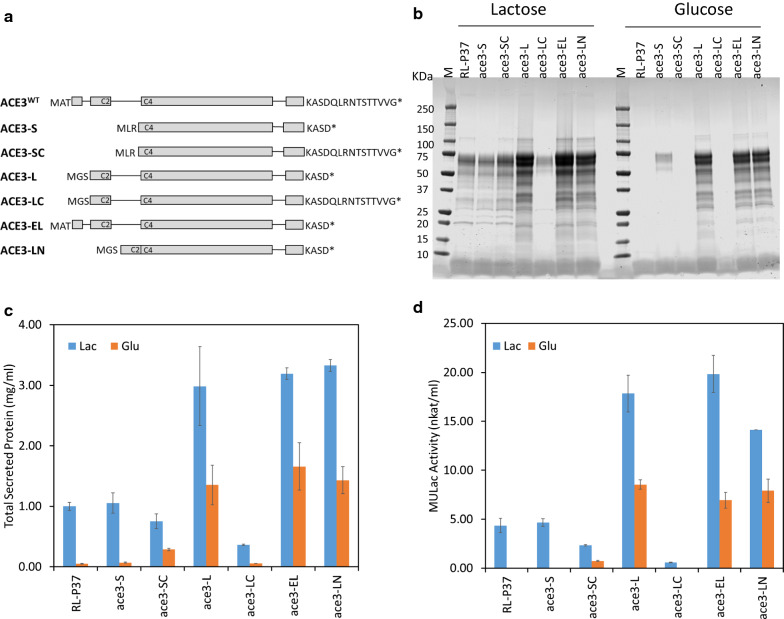
Overexpression of *ace3* variants. **a** Schematic presentation of the different *ace3* variants. Exons are shown as boxes and introns as single lines. The N-terminal and C-terminal amino acid residues are shown. C2 and C4 indicate the presence of 2 and 4 cysteines of the Zn_2_Cys_6_ binuclear domain within exons, respectively. **b** SDS-PAGE gel from culture supernatants. An equal volume of culture supernatant was loaded in each lane. **c** The total secreted protein concentrations as measured by Bradford assays. The mean ± standard deviation was calculated from three biological replicates. **d** MULac activities measured from the culture supernatants. The mean ± standard deviation was calculated from three biological replicates. Lac: lactose; Glu: glucose

To analyze the roles of the different N-termini, C-terminal mutation, and the large intron of the ACE3 transcriptional activator, we expanded the ACE3 variant analysis beyond the currently annotated ones, and constructed 6 *ace3* variants with different combinations of the N- and C-terminus, as well as a deletion of the large intron 2. These different constructs are depicted in Fig. 2a and their corresponding protein sequence alignments and functional domains are shown in Additional file 1: Fig. S2. We did not test the *ace3*^*WT*^ in this work, but included its sequence in figures above for easy comparisons. The *ace3* variants were expressed using a promoter from the *dic1* gene (JGI protein ID: Trire2_47930, encoding a mitochondrial carrier protein, see below) and the expression constructs were integrated at the glucoamylase *gla1* gene locus as a single copy. The protein productivity of the parental and engineered strains expressing different *ace3* variants was evaluated by microtiter plate growth assay, followed by measurement of the total secreted protein, SDS-PAGE gel analysis and cellulase activity assays with MULac as a substrate.

It was evident from the results that the effect on protein production was highly dependent on the *ace3* variant being expressed (Fig. 2b–d). The expression of *ace3*-*L*, *ace3*-*EL* and *ace3*-*LN* (with the large intron 2 deletion) resulted in the highest increase in the amount of total secreted protein compared to the parental strain. In these strains, the amount of total secreted protein when grown with glucose for non-inducing conditions was ~ 30-fold higher than that of the parental strain with the same carbon source, and ~ 1.5-fold higher than that of the parental strain with lactose as the inducing carbon source. These strains also showed ~ threefold higher protein production when grown on lactose compared to the parental strain under the same inducing conditions. Increased cellulase activity on MULac followed a very similar pattern. The protein profile on SDS-PAGE for the *ace3*-*L*, *ace3*-*EL* and *ace3*-*LN* transformants grown on glucose was similar, though not identical, to the parental RL-P37 strain grown on lactose. Some differences between strains could be attributed to differences in glycosylation of secreted proteins. Transformants expressing ACE3-S and ACE3-SC, which both contain an incomplete Zn_2_Cys_6_ domain at N-terminus but differ at their C-termini, did not result in increased protein production in comparison to the parental strain. Interestingly, strain expressing ACE3-LC that comprises an intact Zn_2_Cys_6_ domain at N-terminus and a wild-type C-terminus showed reduced protein production compared to the parental strain.

Taken together, these results suggest that an intact Zn_2_Cys_6_ binuclear cluster domain with all 6 cysteines at N-terminus and the 11-amino acid C-terminal truncation are both required and sufficient to enable high protein secretion on glucose (non-inducing condition), as well as improved production on lactose (inducing condition). The ACE3-L variant contains both required elements, and only marginal, if any, improvement on protein production was observed when its N-terminus was extended (i.e., ACE3-EL), or the large intron was deleted (i.e., ACE3-LN).

### Identification of the ACE3 C-terminal truncations that enable constitutive protein production

The 11-amino acid C-terminal truncation is an SNP that arose from random mutagenesis in the NG14 strain [4]. The C-terminus has been shown to be of importance for ACE3 and some other Zn finger regulators [23, 33]. This led us to further map important residues at the ACE3 C-terminus by generating a set of serial truncations. We used the ACE3-LC variant with an intact Zn_2_Cys_6_ domain at the N-terminus and an intact wild-type C-terminus as the starting molecule, and constructed and evaluated RL-P37 strains ectopically expressing ACE3-LC variants lacking 5, 10, 15, 20 and 25 C-terminal amino acid residues (Additional file 1: Fig. S3). It should be mentioned that the parental strain RL-P37 contains the endogenous copy of *ace3* gene with an 11-amino acid C-terminal truncation. Daughter strains expressing ACE3-LC variants with a 10 or 15 amino acid truncation showed similar improvement in protein production as that with the ACE3-L variant with an 11-amino acid truncation. However, the ACE3-LC variants with 5, 20 or 25 residues removed did not improve secreted protein production on either glucose or lactose. In fact, removal of 20 or 25 residues severely reduced protein production induced by lactose. This result suggests that truncations within a certain area at the ACE3 C-terminus are crucial for high productivity on glucose (non-inducing condition) or lactose (inducing condition).

**Fig. 3:**
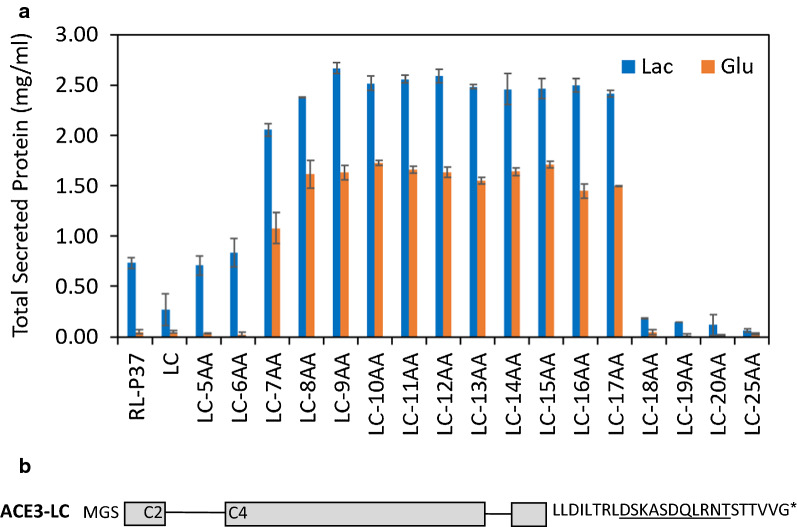
*ace3*-*LC* variants with C-terminal truncations. **a** Total secreted protein concentrations as measured by Bradford assays. Lac: lactose; Glu: glucose. **b** Schematic representation of the ACE3-LC variant protein with a wild-type C-terminus. Exons are shown as boxes and introns as single lines. C2 and C4 indicate the presence of 2 and 4 cysteines of the Zn_2_Cys_6_ binuclear domain within exons, respectively. The first 3 amino acids at the N-terminus and the last 25 amino acids at the C-terminus are shown, and the amino acids at position -17 to -7 are underlined

To further explore the upper and lower limits of permissible ACE3 C-terminal amino acid truncations, we constructed and evaluated RL-P37 strain harboring variants missing from 5 and 20 C-terminal residues with 1 amino acid increments (Fig. 3 and Additional file 1: Fig. S4). Compared to the parental strain RL-P37 grown on lactose, strains expressing an ACE3-LC protein with truncations of 7 to 17 amino acids showed a 1.5 ~ twofold improvement in total protein production when grown on glucose, and they also showed an approximately threefold improvement in total protein production on lactose. In contrast, strains expressing an ACE3-LC protein with either the wild-type C-terminus or a C-terminus with truncations of 5, 6, or more than 17 amino acid produced a minimal amount of protein when grown on glucose, and similar or reduced amount of protein on lactose as compared to the parental strain grown on lactose.

Together these results suggest that whereas overexpression of ACE3-LC with the wild-type C-terminus has little or even a detrimental effect on productivity, overexpression of ACE3-LC with the appropriate C-terminal truncation of 7–17 amino acids can confer to enhanced protein production under both glucose (non-inducing) conditions and lactose (inducing) conditions. Therefore, the ACE3-L variant with an 11-amino acid truncation at C-terminus was selected as the main variant for further study in this work.

### *ace3*-*L* and *ace3*-*LN* can complement an *ace3* deletion

**Fig. 4:**
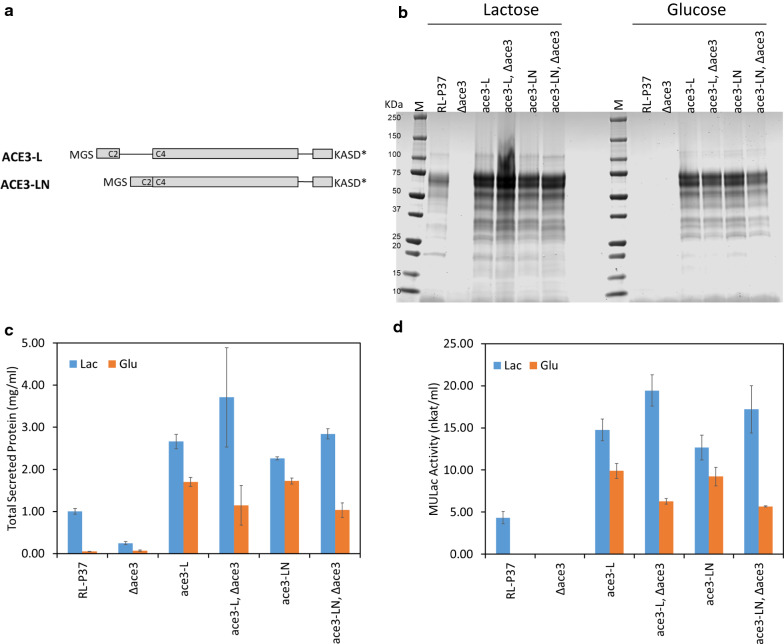
*ace3* deletion. **a** Schematic representation of the different *ace3* variants. Exons are shown as boxes and introns as single lines. The N-terminal and C-terminal amino acid residues are shown. C2 and C4 indicate the presence of 2 and 4 cysteines of the Zn_2_Cys_6_ binuclear domain within exons, respectively. **b** SDS-PAGE gel from culture supernatants. An equal volume of culture supernatant was loaded in each lane. **c** Total secreted protein concentrations as measured by Bradford assays. The mean ± standard deviation was calculated from three biological replicates. **d** MULac activities measured from the culture supernatants. The mean ± standard deviation was calculated from three biological replicates. Lac: lactose; Glu: glucose

To ensure that overexpression of *ace3*-*L* construct alone was sufficient for (hemi)cellulase activation, we complemented an *ace3* deletion strain in RL-P37. For this experiment, two constructs were created: *ace3*-*L* including introns and *ace3*-*LN* where the large intron was removed (Fig. 4a). As shown in Fig. 4a–d, deletion of the *ace3* gene in the RL-P37 strain abolished the production of total secreted proteins on lactose and on glucose, as was expected based on previous studies [20, 21]. However, deletion of the endogenous *ace3* gene from transformants expressing either the *ace3*-*L* variant or the *ace3*-*LN* variant had no clear effect on protein production on either carbon source. These *ace3* delete strains showed either comparable or higher protein production on glucose, and further elevated protein production on lactose when compared to that with the parental strain RL-P37 on lactose. The corresponding cellulase activities on MULac as a substrate exhibited a very similar pattern. Again, the protein profile on SDS-PAGE for the *ace3*-*L* and *ace3*-*LN* transformants grown on glucose was similar, though not identical, to the parental RL-P37 strain grown on lactose. These results suggest that *ace3*-*L* or *ace3*-*LN* constructs expressed ectopically can complement the *ace3* deletion.

### The effects of *xyr1* deletion and overexpression in an *ace3* overexpression strain

XYR1 has been described as a major regulator of cellulose and hemicellulase gene expression. The *ace3* and *xyr1* genes clearly exhibit similar expression patterns under different growth conditions [20–22]. To develop a more complete understanding of the role of *xyr1* in the modified *ace3* strains, we examined the effects of both deletion and overexpression of *xyr1*.

**Fig. 5:**
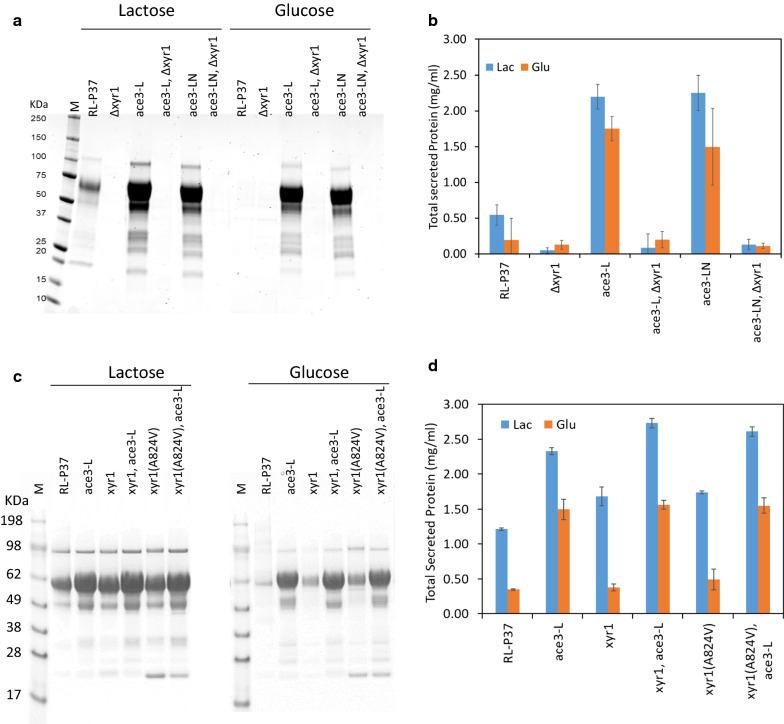
*xyr1* deletion (**a** and **b**) and overexpression (**c** and **d**). **a**, **c** SDS-PAGE gel of culture supernatants. An equal volume of culture supernatant was loaded in each lane. **b**, **d** Total secreted protein concentrations as measured by Bradford assays. The mean ± standard deviation was calculated from three biological replicates. Lac: lactose; Glu: glucose

We first examined the effect of *xyr1* deletion in parental strain RL-P37, and daughter strains expressing *ace3*-*L* or *ace3*-*LN*. As shown in Fig. 5a and b, deletion of the *xyr1* gene dramatically reduced secreted protein production both on lactose and on glucose in all the strains. This result indicated that overexpression of *ace3* was unable to rescue the absence of *xyr1*, and that XYR1 is required for ACE3-dependent activation of cellulase genes.

We next assessed the effect of *xyr1* overexpression in an *ace3*-*L* overexpressing strain. Previously, Derntl et al. [28] showed that a *T. reesei* strain harboring a point mutation (A824V) in XYR1 exhibited elevated expression of xylanases and higher basal expression of cellulases. Here, we included this mutant in our work. The *xyr1*^*WT*^ and *xyr1*^*A824V*^ genes were expressed under the control of a constitutive promoter from the *pdc1* gene (JGI protein ID: Trire2_121534), and the expression cassette was integrated at the *ace1* gene locus, hence inactivating *ace1* gene expression. Previously, Wang et al. [26] showed that combining *xyr1* overexpression and *ace1* down-regulation using RNAi was more effective in boosting total protein production than *xyr1* overexpression alone. As shown in Fig. 5c and d, strains overexpressing *xyr1*^*WT*^ or *xyr1*^*A824V*^ showed similar improvement in total secreted protein on both lactose and glucose. Compared to RL-P37 grown on the same carbon source, both strains increased total secreted protein by ~ 40% on lactose and by 10% on glucose. Compared to the *ace3*-*L* overexpression strain grown on the same carbon source, the additional overexpression of *xyr1*^*WT*^ and *xyr1*^*A824V*^ improved protein production by ~ 15% on lactose and by ~ 5% on glucose. These results suggest that overexpressing *xyr1* could provide a modest boost to protein production under inducing conditions.

We also examined the growth of these strains on Vogel’s agar plates with glucose as the sole carbon source (Additional file 1: Fig. S5). Overexpression of *xyr1*^*W*T^, *xyr1*^*A824V*^ or *ace3*-*L* alone did not impact cell growth. However, the co-expression of *xyr1* and *ace3*-*L* impaired cell growth and reduced colony size on Vogel’s agar plates. This growth defect was more severe in the strain co-expressing *xyr1*^*A824V*^ and *ace3*-*L* than in the strain co-expressing *xyr1*^*WT*^ and *ace3*-*L*. We tested strains with *xyr1* and *ace3*-*L* driven from weaker promoters than the *pdc1* and *dic1* promoters, respectively, but no improvement in protein production or cell fitness was found (data not shown). It should be noted that the *ace1* gene was disrupted in these overexpression strains. Previous work showed that deletion of the *ace1* gene resulted in reduced cell growth on agar plate with Solka floc cellulose as the sole carbon source or in liquid minimal medium with sorbitol, whereas cell growth on glucose was not notably affected [16, 34]. We found that the growth of strains with *xyr1* or *xyr1*^*A824V*^ integrated at *ace1* locus was similar to the parental strain RL-P37 both on glucose (Additional file 1: Fig. S5) and on lactose (data not shown). Although we cannot completely rule out that the *ace1* gene disruption has a role in cell growth under our growth conditions, the observed growth defect is likely due to the co-expression of *xyr1* and *ace3*-*L*. We conclude that a slight improvement in production by overexpressing *xyr1* in the *ace3*-*L* background can be compromised by growth defects of the strain.

### Optimization of *ace3* expression level on glucose in an industrial strain

Next, we evaluated the benefit of overexpressing the *ace3*-*L* variant in a proprietary industrial strain T4abc, a high productivity mutant derived from strain RL-P37. We initially used constitutive promoters of the *hxk1* and *pki1* genes to drive expression of *ace3*-*L* and observed a slow growth phenotype. Cell growth rates in liquid NREL media were reduced to 78% with the strain harboring the *P*_*hxk1*_-*ace3*-*L* expression construct and to 53% with the strain harboring the *P*_*pki1*_-*ace3*-*L* construct compared to the parental strain T4abc under the same growth conditions in a BioLector microbioreactor (Additional file 1: Fig. S6A). We postulated that this impaired growth phenotype was due to the constitutively high expression of *ace3*-*L* during growth phase.

To maximize protein production and minimize negative effects on growth, we screened 18 different promoters to drive expression of *ace3*-*L*. The promoters that showed minimal expression during the initial growth phase with batched glucose and various expression levels (ranging from low to high during production phase at a slow feed rate with glucose) were selected. The promoter-*ace3*-*L* expression constructs were introduced in strain T4abc and their impacts on cell growth rate and protein production were evaluated using light scattering as measured by the BioLector microbioreactor apparatus and total protein assays, respectively. Most of the tested promoters showed improved protein production on glucose, albeit to various degrees. Interestingly, the native *ace3*-*EL* promoter showed the lowest improvement, and the *dic1* promoter showed the highest total secreted protein with minimal impact on cell growth rate (Table 1, and Additional file 1: Fig. S6B). The *dic1* promoter was hence used as the promoter of choice in this study.

**Table 1:** Total secreted protein titers of *T. reesei* parental strain T4abc and engineered strains expressing *ace3*-*L* driven from different promoters under inducing and non-inducing conditions

Strain ID	Promoter (protein ID^1^)	Glu/Sop^2^	Glu^3^
T4abc (parental)	N/A	1.00	0.20
LT82	*OPT (44278)*	1.16	1.07
LT83	*dic1(47930)*	1.35	1.12
LT85	*gut1 (58356)*	0.95	1.08
LT86	*hxk1 (73665)*	0.96	0.73
LT87	*pki1 (78439)*	1.16	0.98
LT149	*rev3 (103041)*	0.96	0.76
LT150	*Trire2_104295*	0.94	0.41
LT151	*tkl1 (2211)*	1.02	1.01
LT152	*bxl1 (121127)*	1.00	1.04
LT154	*dld1 (5345)*	1.00	0.58
LT155	*xyn4 (111849)*	0.95	0.95
LT156	*glr1 (72526)*	0.95	0.89
LT157	*axe1 (73632)*	1.01	0.85
LT158	*ace3EL (77513)*	1.02	0.29
LT242	*Trire2_69944*	1.00	0.82
LT246	*Trire2_67752*	1.00	0.74
LT252	*Trire2_3739*	1.01	0.87
LT258	*Trire2_2499*	1.05	0.79

Protein ID^1^ is based on JGI QM6a annotation (genome.jgi.doe.gov/Trire2/Trire2.home.html)

Glu/Sop^2^ is an abbreviation of “Glucose/Sophorose”; inducing condition

Glu^3^ is an abbreviation of “Glucose”; non-inducing condition

Relative protein concentration with the parental strain on Glu/Sop (set as 1) is shown. The numbers are the average from at least three biological replicates

### Overexpression of *ace3*-*L* improves protein production in an industrial strain

**Fig. 6:**
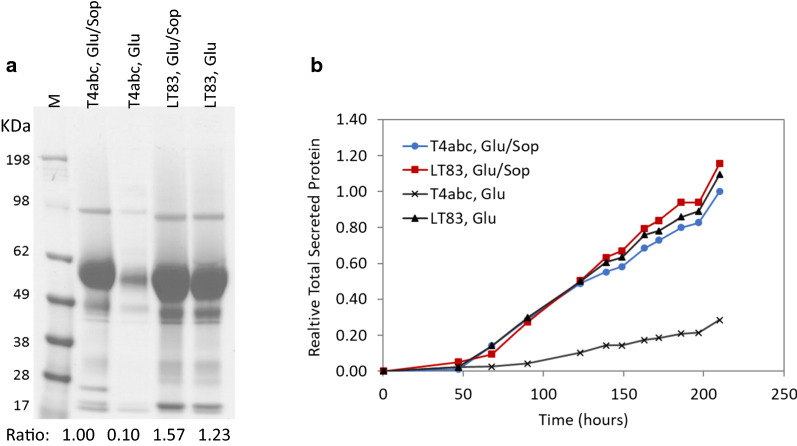
Overexpression of *ace3*-*L* improves protein production in an industrial strain. **a** SDS-PAGE of culture supernatants from *T. reesei* parental strain T4abc and its daughter strain LT83 (expressing *ace3*-*L*). An equal volume of culture supernatant was loaded in each lane. The total secreted protein concentration of each strain (as measured by Bradford assays) was shown as relative ratio to that of strain T4abc grown glucose/sophorose (set at 1). Glu/Sop, glucose/sophorose; Glu, glucose; M, protein molecular weight marker. **b** Protein production of *T. reesei* parental strain T4abc and daughter strain LT83 in 2-L fermenters. The total protein produced by the parental strain fed with glucose/sophorose at the end of fermentation was arbitrarily set at 1, and the relative amounts of protein produced by each strain at each time points were plotted

We assessed the benefit of overexpressing *ace3*-*L* in the industrial strain both in shake flasks and in 2-L bioreactors. When grown in shake flasks, the parental *T. reesei* cells produced a high amount of secreted proteins only in the presence of the sophorose inducer. In shake flask cultures, the engineered strain LT83 (expressing *ace3*-*L* under the control of the *dic1* promoter) secreted increased amounts of protein, with a ~ 50% increase under glucose/sophorose fed (inducing) and ~ 25% under glucose fed (non-inducing) conditions compared to that produced by the parental strain under glucose/sophorose fed (inducing) condition (Fig. 6a). Similar results were observed during fermentation in 2-L bioreactors (Fig. 6b). The parental *T. reesei* strain produced only basal level of protein under non-inducing conditions but secreted a large amount of proteins in the presence of the sophorose inducer. The engineered strain LT83 produced increased amounts of protein, under both inducing and non-inducing conditions compared to the parental strain under inducing conditions, i.e., ~ 20% and ~ 10% improved yields, respectively. Together these results suggest that overexpression of *ace3*-*L* improved protein production in the industrial strain both in the absence and in the presence of an inducer, the latter showing slightly higher titers. We noted that improvements observed with the industrial proprietary strain expressing *ace3*-*L* were lower than that with the public strain RL-P37 expressing the same variant. This discrepancy is not uncommon when using a more advanced industrial strain lineage.

To characterize the cellulase and hemicellulase expression patterns of ACE3-L strains, time-course samples of the parental host T4abc and strain LT83 expressing *ace3*-*L* were taken during fermentation and their gene expression profiles were analyzed using RNA-seq. The relative expression levels of major cellulase and hemicellulase genes obtained by RNA-seq after normalization are shown in Additional file 1: Fig. S7. Similar expression patterns were observed with strain LT83 fed with glucose (non-inducing), glucose/sophorose (inducing), and strain T4abc fed with glucose/sophorose (inducing). The cellulase and hemicellulase expression levels were lower in T4abc strain fed with glucose (non-inducing) condition, which is consistent with total protein measurement result. This result suggests that overexpression of *ace3*-*L* does not alter the relative expression pattern of major cellulases and hemicellulases.

## Discussion

ACE3 is an essential transcription factor for cellulolytic enzyme expression in *T. reesei*. In this study, we dissected the *ace3* gene structure and identified critical regions required for its function. When expressed in the appropriate form and at an appropriate level, ACE3 enabled protein production in the absence of an inducer and improved protein production in the presence of an inducer.

The initial study on ACE3 by Häkkinen et al. [20] tested overexpression of a QM6a annotated version of ACE3. At the time of that work this was the only annotated version available, containing an incomplete Zn_2_Cys_6_ binuclear cluster domain with only 4 cysteine residues. Recently, Zhang et al. [21] showed that the QM6a-annotated ACE3 variant was unable to bind DNA and identified the “complete” ACE3 wild-type comprising a Zn_2_Cys_6_ domain with 6 cysteine residues. In this study, we expanded the ACE3 N-terminal variants to three different versions, namely ACE3-SC, ACE3-L and ACE3-EL (Fig. 2 and Additional file 1: Fig. S2). The ACE3-SC variant, comprising a truncated DNA binding domain, is the same as was used in the study by Häkkinen et al. [20] and the “nominal Ace3” of Zhang et al. [21]. The ACE3-L variant is based on the annotation in the RUT-C30 strain genome (JGI). The ACE3-EL variant was identified by sequencing of a cDNA clone of *ace3*, and shared the same N terminal sequence with wild-type ACE3 described in [21]. The predicted L and EL proteins both contain the full-length Zn_2_Cys_6_ DNA-binding domain (the EL version is 45 amino acids longer at the N-terminus), suggesting that these variants would be able to bind to the promoters of target genes. We showed that either the *ace3*-*EL* or *ace3*-*L* gene variants were able to improve production of total secreted proteins on glucose and on lactose (Fig. 2). Total protein production in the *ace3*-*SC* expressing strain was similar to that of the parental strain, corroborating the data of Zhang et al. [21] which indicates that the incomplete Zn_2_Cys_6_ is not sufficient to drive cellulase production. These data support the hypothesis that direct binding of ACE3 to the promoter regions of its target genes is important for the induction of the (hemi)cellulolytic enzymes. There is an unusually long intron (i.e., intron 2) in the *ace3* gene that is over 400 bp long and as such is atypical for ascomycete fungi, where introns are generally below 100 bp [35]. We identified a sophorose-inducible promoter nested within this long intron. Our 5′-RACE analysis to map *ace3* transcription start sites (TSSs) revealed two major transcript initiation regions. The *ace3*-*EL* transcript started upstream of exon 1 and the *ace3*-*SC* transcript started within the long intron 2, ~ 148 bp upstream of the presumptive start codon of the *ace3*-*SC* ORF. Supporting this, RNA-seq data and RT-qPCR results showed different expression levels of *ace3*-*EL* and *ace3*-*SC* transcripts under different growth conditions, where the *ace3*-*EL* transcript was moderately induced by sophorose and *ace3*-*SC* transcript was strongly induced by sophorose. It should be noted that we have no evidence that the *ace3*-*SC* transcripts are translated and whether they have any functional roles. More data will be needed to elucidate a potential role of this short *ace3*-*SC* transcript or its encoded protein upon induction.

We found that a C-terminal truncation of ACE3 is essential for cellulase production on glucose. Derivative strains of NG14, like RUT-C30 and RL-P37, harbor a point mutation, not seen in QM6a, resulting in the loss of 11 amino acids at the ACE3 protein C-terminus. This truncation was recently shown to be a crucial mutation for elevated cellulase expression in the aforementioned strains [23]. We verified this mutation in RL-P37 by sequencing and constructed strains overexpressing either the wild-type C-terminus in the SC and LC variants or the 11 amino acid-truncated C-terminus in all the other variants (Fig. 2, Additional file 1: Fig. S2). Overexpression of the ACE3-LC variant with a complete Zn_2_Cys_6_ DNA-binding domain at the N-terminus and the wild-type C-terminus had only minor effects on the production of secreted proteins. In contrast, all ACE3 variants harboring specific truncations at their C-termini had a profound impact on secreted protein production (Fig. 3). Zhang et al. [21] showed that ACE3 contains an activation domain at the C-terminus and suggested that ACE3 can form either homo- or hetero-dimers with XYR1 through their C-termini. Here, we showed that truncations of 7 to 17 amino acids at C-terminus are essential for improved protein production. Our findings suggest that there is a so far unidentified inhibitory domain at the very end of the C-terminus of the ACE3 protein that is functionally distinct from the previously identified dimerization domain. Alternatively, the C-terminal region also contains a short stretch of hydrophobic residues that may interact with other partners to repress transcription. By shortening the C-terminus, we may disrupt such an interaction. Several fungal Zn_2_Cys_6_ transcription factors with a similar structural organization have been shown to harbor a repressor domain at C-terminus [33]. For example, in *Saccharomyces cerevisiae,* a C-terminal domain in the Gal4p transcriptional factor interacts with an inhibitory protein Gal80p [36]. Pdr1p, another Zn_2_Cys_6_ transcription factor from *S. cerevisiae*, was proposed to have two configurations: an activated open form and an inhibited closed form. In the closed form, the C-terminal inhibitory domain of the protein masks the activation domain and a point mutation releases it from the inhibition [37]. In *Aspergillus niger* XlnR, a single amino acid mutation (V756F) resulted in xylanase production under repressing conditions. A d-glucose inhibition domain in the C-terminal region was proposed to respond to repressing signals and mask the activation domain in XlnR [30]. Based on our results, the C-terminal 17 amino acids of ACE3 could be a part of a repressor domain itself or of a domain that interacts with a repressor, which is deactivated by the 11-amino acid truncation in RUT-C30 and RL-P37 strains. Our data also suggest that deletion of more than 17 amino acids from the C-terminus creates an inactive ACE3, presumably due to interference with another essential domain or formation of a misfolded protein.

Subcellular location and transportation across the nuclear membrane play an important role in how and when a transcriptional factor interacts with its target gene(s) [30, 38]. We found two putative nuclear location signals (NLS) at the ACE3 N-terminus and at least one leucine-rich nuclear export signal (NES) at its C-terminus (Additional file 1: Fig. S2). Truncation of 17 amino acids at the C-terminus removes the last two residues of the putative NES, while truncation of 18 amino acids removes the most conserved leucine in this motif and may impair correct translocation of ACE3. Further study is required to understand the precise role of the ACE3 C-terminus.

When comparing our findings with those of other authors, it should be taken into account that we used RL-P37 as a host strain and overexpressed *ace3* variant genes from an ectopic site. Therefore, the transformants produced a mix of the overexpressed ACE3 variant protein in addition to native ACE3 with an 11-amino acid long truncated C-terminus. Häkkinen et al. [20] used strain QM9414 and overexpressed ectopically the *ace3*-*SC* variant with wild-type C-terminus in addition to the native *ace3*, also with wild-type C-terminus. In contrast to our data, they showed that there was some improvement in secreted enzyme production under lactose-inducing conditions when the *ace3*-*SC* variant with wild-type C-terminus was overexpressed. This is difficult to reconcile with the understanding that this ACE3-SC variant would not have a functional Zn_2_Cys_6_ DNA-binding domain. A possible explanation is that the ACE3-SC variant has a functional C-terminal repressor-binding region that could titrate a hypothetical repressor allowing for a slightly higher induction by the native ACE3 protein. In another study, Zhang et al. [21] used a promoter swap strategy at the *ace3* locus in strain QM6a to overexpress two *ace3* variants in the absence of its native *ace3*, the *ace3*-*SC* variant and the wild-type *ace3* that shared the same N-terminal sequence with *ace3*-*EL* but with the wild-type C-terminus. They observed increased enzyme productivity when overexpressing the wild-type *ace3* under inducing conditions with Avicel, suggesting that a higher concentration of functional ACE3 enhanced induction under inducing conditions. The different results observed in other studies as compared to those presented here could be explained by the difference in a host strain, *ace3* expression strategy and/or growth conditions.

From our results and those of others, it is apparent that *xyr1* and *ace3* genes are co-regulated and that XYR1 and ACE3 operate cooperatively during (hemi)cellulase gene induction. XYR1 protein was shown to be synthesized de novo at the onset of induction [38]. Our qPCR analysis indicated that *xyr1* expression is induced in the *ace3*-*L* expressing strain on glucose, but the induction was not as clear on lactose (results not shown). This is likely because lactose is a strong inducer for *xyr1* expression, masking the induction effect from ACE3-L. Induction in the presence of glucose mediated by overexpression of *ace3* does not overcome carbon catabolite repression. We have observed that (hemi)cellulase production does not occur in culture with a high glucose concentration, and only occurs after glucose has been depleted below a certain threshold (results not shown).

Overexpression of *xyr1* has been observed to enhance cellulase production, using the wild-type *xyr1* [39] or a mutated *xyr1* variant [28, 29], or a hybrid transcriptional factor comprising the DNA-binding domain of XYR1 and the transactivation domain of another transcriptional factor [31, 40]. Xue et al. [41] recently showed that by randomly integrating copies of constitutively expressed *xyr1* and *ace3* in *Trichoderma orientalis*, cellulase production was improved in media with a cellulosic inducer or with glucose. In our study, both *ace3* and *xyr1* can, when overexpressed alone, improve the expression of their target genes, but only modest additive effect was observed when both genes were overexpressed in *T. reesei* strains. It should be noted that the ACE3 used in *T. orientalis* was homologous to the ACE3-SC variant comprising the truncated Zn_2_Cys_6_ DNA-binding domain and the wild-type C-terminus. Hence, the induction mechanism may be different in the two systems. Both *xyr1* and *ace3*-*L* were expressed ectopically in our co-expression strains, which could upregulate the auto-regulatory loop of the native *xyr1* gene. In a *xyr1*^*A824V*^ overexpression strain, both the native and mutant XYR1 co-exist which could further interfere with the regulatory network of XYR1. Further investigation would be needed to optimize the co-expression system of ACE3 and XYR1 to maximize its potential benefit in improving protein production.

## Conclusions

In this work, we showed that overexpression of an *ace3* variant with intact Zn_2_Cys_6_ DNA-binding domain and a C-terminus truncated by 7–17 amino acids led to improved (hemi)cellulase production under inducing conditions in strain RL-P37 and its derivative production strain T4abc. More importantly, productivity in the presence of glucose without added inducer was at levels comparable to, or higher than, those observed with the parental strain under inducing conditions. To our knowledge, this is the first report to show that overexpression of the ACE3 transcriptional factor alone can improve productivity under non-inducing conditions. The mechanism by which this constitutive production occurs is not clear, but it mimics very closely induction in the parental strain by lactose or sophorose. Relative levels of individual secreted enzymes produced by strains overexpressing the *ace3*-*L* variant under either inducing or non-inducing conditions were very similar to the product of the parental strain under inducing conditions. Hence, the strains do not need any additional modifications to produce an optimal enzyme composition for biomass degradation. ACE3 overexpression also improves the expression of heterologous proteins under control of the *cbh1* promoter (data not shown). In general, the described approach can be applied as a universal method for achieving high levels of expression in the filamentous fungus *T. reesei* without an inducer.

## Materials and methods

### Strains and cultivation conditions

*Trichoderma reesei* strain RL-P37 (NRRL Deposit No. 15709), a hyper-cellulolytic strain, was used as a parental strain in this study [4]. RL-P37 was developed from the wild-type *T. reesei* strain QM6a (ATCC Deposit No. 13631) via several random mutagenesis steps and was modified to inactivate the *pyr4* gene. T4abc is a proprietary industrial strain derived from strain RL-P37. *T. reesei* parental strains and their daughter strains used in this study are listed in Additional file 2: Table S1. *T. reesei* strains were routinely maintained on Vogel’s agar with 1% glucose [42] at 28 °C for 5–7 days with alternate light/dark cycle (12 h light: 12 h dark).

For protein expression microtiter plate (MTP) assays, approximately 10^5^ spores were inoculated into 2 mL liquid NREL medium in a deep-24-well microtiter plate. The NREL medium was previous described in [43]. The NREL medium was supplemented with 2% glucose (weight/volume, non-inducing condition) or 2% lactose (weight/volume, inducing condition) as the sole carbon source. The cultures were incubated at 28 °C, 250 rpm, and 85% humidity for 5 days.

For protein expression shake flask assays, approximately 10^6^ spores were added to 50 mL of YEG broth in a 250-mL Erlenmeyer flask with bottom baffles. The YEG broth contains 5 g/L yeast extract and 22 g/L glucose. The cell cultures were grown for 48 h, followed by sub-culturing into fresh YEG for another 24 h. These seed cultures were then inoculated into either 50 mL of NREL medium supplemented with either 2% glucose (non-inducing condition) 2% glucose/sophorose (a mix of glucose, sophorose and other transglycosylation products created by the action of cellulase on glucose, inducing condition) [25], or 2% lactose (inducing condition) in 250-mL shake flasks with bottom baffles. All shake flasks were incubated at 28 °C with continuous shaking at 200 rpm.

For fed-batch fermentation, the strains were fermented generally as previously described [25]. In brief, the fermentation was performed in a 2-L bioreactor under a two-phase cultivation procedure: cells were first grown in minimal medium containing 75 g/L of glucose as carbon source. When the initial glucose was close to depletion, a fed phase (i.e., production phase) was initiated. Cells were fed with either glucose or glucose/sophorose. Samples were collected periodically to determine biomass, glucose and protein concentration.

### Vector and strain constructions

The vectors used in this study are listed in Additional file 2: Table S2. The overexpression vectors were constructed using shuttle vector pRS426 as the backbone and yeast recombination cloning as the assembly method [44, 45]. These vectors were designed to enable targeted integration of *ace3* expression cassettes at the *gla1* locus (JGI protein ID: Trire2_1885) and *xyr1* expression cassettes to the *ace1* locus (JGI protein ID: Trire2_75418) in *T. reesei*. The *ace3* variant overexpression vectors harbor an approximately 1.5 kb 5′ homology flank region and a 1.5 kb of 3′ homology flank region of *gla1* ORF needed for targeted integration. The *ace3* expression vectors also generally include an approximately 2 kb *T. reesei dic1* promoter sequence operably linked to the *ace3* ORF coding sequence with the native *ace3* gene terminator of approximately 500 bp. The *xyr1* overexpression vectors have an approximately 1.5 kb 5′ homology flank region and a 1.5 kb of 3′ homology flank region of *ace1* ORF needed for targeted integration. The *xyr1* expression vectors include an approximately 1.5 kb *T. reesei pdc1* promoter sequence operably linked to the *xyr1* ORF coding sequence with the native *xyr1* gene terminator of approximately 800 bp. The 5′ and 3′ flanks of the *T. reesei* genes needed for targeted integration, the promoters, the different *ace3* variants and *xyr1* coding regions and terminators were produced by PCR using primers listed in Additional file 2: Table S3. Template for the flanking fragments was genomic DNA from wild-type *T. reesei* QM6a. Fragments for constructing the different *ace3* variants were amplified from either *T. reesei* QM6a- or RUT C-30-strain genomic DNA. For deletion of the *xyr1* and *ace3* gene, the 5′- and 3′-flanking regions of the gene were amplified from QM6a genomic DNA. For the deletion vectors, approximately 1.5-kb fragments of both the 5′ homology flank regions and 3′ homology flank regions of *ace3* or *xyr1* ORF were created by PCR. *T. reesei pyr4* marker gene was cloned in between the flanks using shuttle vector pRS426 as the backbone and yeast cloning as the assembly method [44, 45].

The expression vectors were digested with *PmeI* to release the fragments for targeted integration and separated with agarose gel electrophoresis. Correct fragments were isolated from the gel using a Qiagen gel extraction kit (Qiagen, MA; Cat No. 28704) according to the manufacturer’s protocol. Approximately, 10-µg purified fragment was used to transform protoplasts of a *pyr4*^−^ mutant of *T. reesei* RL-P37 strain. The transformation was performed using the polyethylene glycol (PEG)-mediated protoplast transformation protocol [43, 46]. The correct transformants obtained by homology recombination were verified by diagnostic PCR using primers listed in Additional file 2: Table S3. The strains were verified by Southern blot analysis. DNA for Southern blot analysis was purified with Easy-DNA kit for genomic DNA isolation (Invitrogen; Cat. No: K180001), according to the manufacturer’s instructions. Southern blot analysis was performed according to the protocol for homologous hybridizations in Sambrook et al. [47] using radioactive labeling (^32^P-dCTP) and DecaLabel Plus kit (Thermo Fischer Scientific; Cat. No. K0622) (data not shown).

### 5′-RACE and RT-qPCR

Rapid Amplification of 5′-cDNA Ends (5′-RACE) was performed using the FirstChoice RLM-RACE kit (ThermoFisher Scientific, MA; Catalog No. AM1700) according to the manufacturer’s protocol. Two gene-specific reverse primers S-R2 (for outer PCR) and S-R3 (for inner PCR) were used with forward primers from the kit (Additional file 2: Table S3). Both gene-specific primers anneal to the 5′ region of exon3. Reverse transcriptions and 5′-RACE reactions were performed in quadruplicate followed by sequencing of individual reaction products.

The real-time reverse-transcription quantitative polymerase chain reaction (RT‑qPCR) was performed as previously described [48]. In brief, the total RNA was isolated from *T. reesei* strains using Qiagen RNAeasy (Qiagen, USA. Catalog No. 74106). Reverse-transcription (RT) was performed using a High Capacity cDNA Archive Kit (Applied Biosystems, Foster City, CA, USA. Catalog No. 4368814). Real‐time analysis was performed using TaqMan Universal PCR Master Mix from Applied Biosystems, using forward and reverse primers and probes listed in Additional file 2: Table S3. Real‐time PCR primers and probes were designed using Primer Express Software v 2.0 (Applied Biosystems). The transcriptional level of a house-keeping gene *act1* was used for reference calculation and data were normalized to the transcriptional level of *ace3* exon 1 at early growth phase.

### Bradford assays and SDS-PAGE

The secreted protein concentrations were measured in microtiter plates with Bradford protein reagent concentrate using bovine γ-globulin as standard (Bio-Rad, Hercules, CA, USA). Ten microliters of sample and 200 μL of the 1:5 diluted reagent were pipetted into wells of 96-well plates and the absorbance at 595 nm was measured 5–30 min after the addition of reagent with Varioskan Flash (ThermoFisher Scientific, MA).

Protein samples were separated under reducing conditions on NuPAGE 4–12% Bis–Tris gel (ThermoFisher Scientific, MA). Gels were stained with SimplyBlue SafeStain (ThermoFisher Scientific, MA) according to manufacturer’s instructions.

### Cellulase activity assays

Cellulase activities were measured via assaying 4-methylumbelliferyl-β-d-lactoside (MULac) (Carbosynth, Compton, Berkshire, UK) cleavage. Samples were diluted to 50 mM Na-acetate (pH 5.0) and 50 μL were pipetted to the wells of black microtiter plate. Reaction was started by the addition of 50 μL 1 mg/mL MULac (~ 2 mM) and stopped 15 min later by the addition of 100 μL 1 M sodium carbonate. Fluorescence was measured with a Varioskan Flash (ThermoFisher Scientific, MA) with 355/460 nm excitation and emission wavelengths. The standard curve ranged from 1.25 to 40 μM 4-methylumbelliferyl.

Additional materials and methods, including RNA-sequencing and data analysis, protein sequence alignment and motif searches, and growth curve with BioLector, are described in Additional file 2.

## Supplementary information

**Additional file 1: Figure S1.** Identification of transcriptional start sites (TSS) at *ace3* locus. **Figure S2.** Protein sequence alignment of different ACE3 variants. **Figure S3**. ACE3-LC variant with C-terminus truncation at a 5-amino acid increment. **Figure S4.** SDS-PAGE with strains overexpressing ACE3-LC variant with C-terminus truncation. **Figure S5.** Growth of the parental strain (RL-P37) and its daughter strains overexpressing *xyr1* and/or *ace3* variants on Vogel’s plate at 28 °C for 5 days, with an alternate light/dark cycle (12 h light:12 h dark). **Figure S6.** Growth of the parental T4abc and its daughter strains expressing the *ace3*-*L* variant driven by a *hxk1* promoter or a *pki1* promoter (A), and the daughter strain with *ace3*-*L* expression driven from a *dic1* promoter (B). **Figure S7.** Heat map visualization of expression data on the genes encoding cellulases and hemicellulases in parental strain T4abc and engineered strain LT83 expressing *ace3*-*L.*

**Additional file 2: Table S1.** Strains used in this study. **Table S2**. Plasmid vectors used in this study. **Table S3**. Oligonucleotide primers used in this study.

**Additional file 3:** Supplemental materials and methods. The materials and methods include RNA-sequencing and data analysis, protein sequence alignment and motif search, and growth curve with BioLector.

## Data Availability

The datasets generated and/or analyzed during the current study are not publicly available due to proprietary reasons, but are available from the corresponding author on reasonable request.

## Abbreviations

ACE3: Activator of cellulase expression 3
XYR1: Xylanase regulator 1
AA: Amino acid
PCR: Polymerase chain reaction
MULac: 4-Methylumbelliferyl-β-d-lactoside
NLS: Nuclear localization signal
NES: Nuclear export signal
UTR: Untranslated region
TSS: Transcriptional start site
kDa: KiloDalton
SNP: Single nucleotide polymorphism
