# Supplementary material for: Modification of transcriptional factor ACE3 enhances protein production in Trichoderma reesei in the absence of cellulase gene inducer
Source: Biotechnol Biofuels. 2020 Aug 6;13:137. doi: 10.1186/s13068-020-01778-w (PMC7412840; doi:10.1186/s13068-020-01778-w)
Supplement: Supplementary file 1 — Additional file 1: Figure S1. Identification of transcriptional start sites (TSS) at ace3 locus. Figure S2. Protein sequence alignment of different ACE3 variants. Figure S3. ACE3-LC variant with C-terminus truncation at a 5-amino acid increment. Figure S4. SDS-PAGE with strains overexpressing ACE3-LC variant with C-terminus truncation. Figure S5. Growth of the parental strain (RL-P37) and its daughter strains overexpressing xyr1 and/or ace3 variants on Vogel’s plate at 28 °C for 5 days, with an alternate light/dark cycle (12 h light:12 h dark). Figure S6. Growth of the parental T4abc and its daughter strains expressing the ace3-L variant driven by a hxk1 promoter or a pki1 promoter (A), and the daughter strain with ace3-L expression driven from a dic1 promoter (B). Figure S7. Heat map visualization of expression data on the genes encoding cellulases and hemicellulases in parental strain T4abc and engineered strain LT83 expressing ace3-L. [file 13068_2020_1778_MOESM1_ESM.pdf]

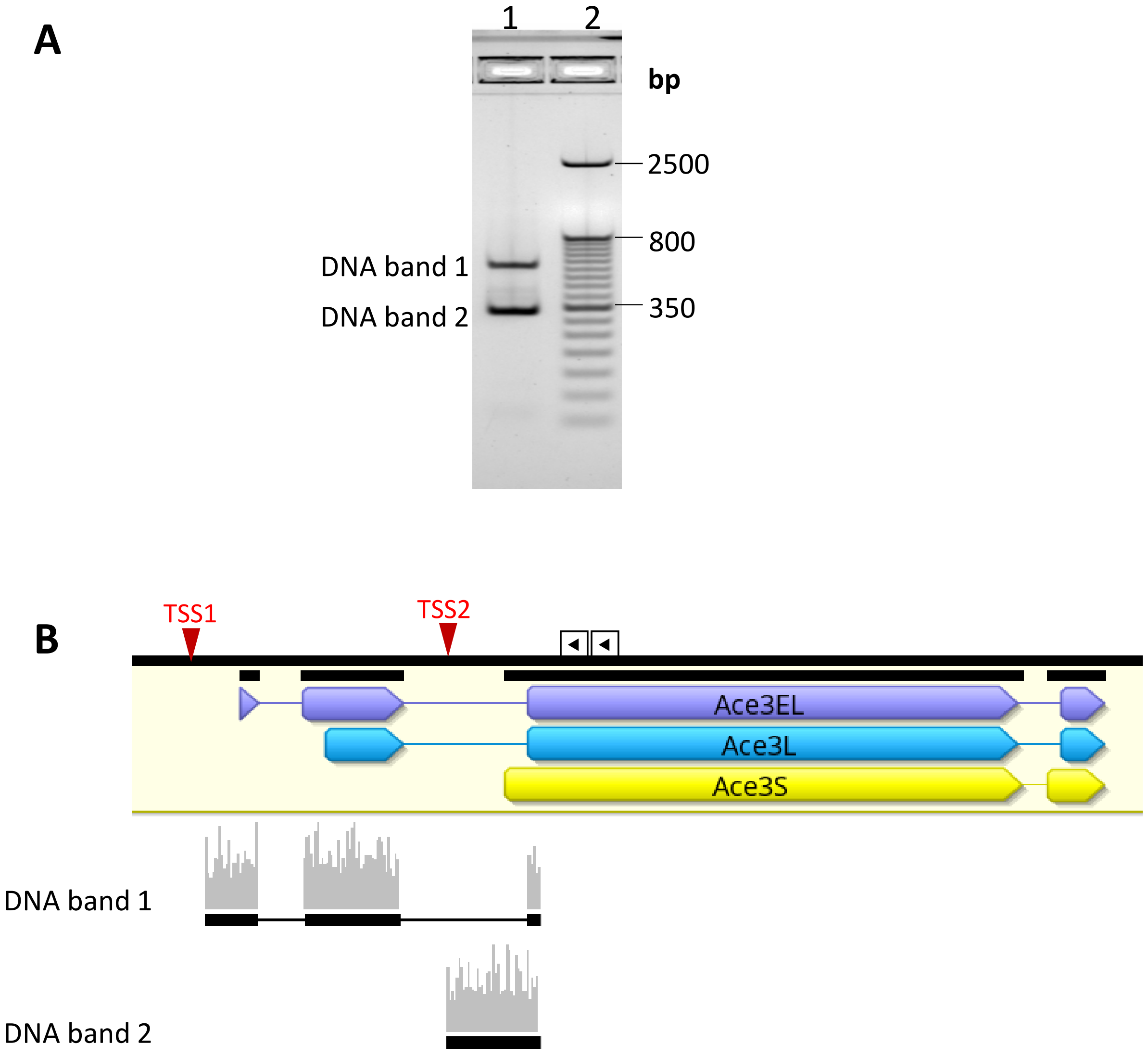

Figure S1. Identification of transcriptional start sites (TSS) at *ace3* locus. **(A)** Results of 5'-RACE experiments. DNA sequences were detected by gel electrophoresis using 2% agarose. Lane 1, two DNA bands were detected, one is ~320 bp, the other is ~580 bp; Lane 2. DNA molecular weight marker. **(B)** Sequences of the two DNA bands were mapped the *ace3* locus. TSS1 is located at a position ~78bp upstream of the start codon of the *ace3-EL* ORF; TSS2 is located at ~148bp upstream of the presumptive start codon of the *ace3-S* ORF. ▼, Transcriptional start sites (TSS). ◄, The annealing sites of gene-specific reverse primers used in 5'-RACE.

```
              1         10        20        30        40        50        60
              |         |         |         |         |         |         |
ace3-WT    MATAAAAAAAGGAAVAAGADTGAAGSSSTGPPGLPGLPGTRTGSVAMGSAAPAQGSVAAAA
ace3-EL    MATAAAAAAAGGAAVAAGADTGAAGSSSTGPPGLPGLPGTRTGSVAMGSAAPAQGSVAAAA
ace3-L     ---------------------------------------------MGSAAPAQGSVAAAA
ace3-LN    ---------------------------------------------MGSAAPAQGSVAAAA
ace3-LC    ---------------------------------------------MGSAAPAQGSVAAAA
ace3-S     ------------------------------------------------------------
ace3-SC    ------------------------------------------------------------

ace3-WT    GGPPAAGAGAGAGAVHALTTSPESASASQPGSPTASTTPPQNSLVSAATSFHHHPRGRLVSR
ace3-EL    GGPPAAGAGAGAGAVHALTTSPESASASQPGSPTASTTPPQNSLVSAATSFHHHPRGRLVSR
 ace3-L    GGPPAAGAGAGAGAVHALTTSPESASASQPGSPTASTTPPQNSLVSAATSFHHHPRGRLVSR
ace3-LN    GGPPAAGAGAGAGAVHALTTSPESASASQPGSPTASTTPPQNSLVSAATSFHHHPRGRLVSR
ace3-LC    GGPPAAGAGAGAGAVHALTTSPESASASQPGSPTASTTPPQNSLVSAATSFHHHPRGRLVSR
ace3-S     -----------------------------------------MLRYSPVLHL----DTLSL
ace3-SC    -----------------------------------------MLRYSPVLHL----DTLSL

                Putative NLS                                Putative NLS
ace3-WT    ACDRCRRRKAKCEYLSAVDSCTHCRDAHVQCTFDLPLARRGPKARKKSDQPGQPPPDPSS
ace3-EL    ACDRCRRRKAKCEYLSAVDSCTHCRDAHVQCTFDLPLARRGPKARKKSDQPGQPPPDPSS
 ace3-L    ACDRCRRRKAKCEYLSAVDSCTHCRDAHVQCTFDLPLARRGPKARKKSDQPGQPPPDPSS
ace3-LN    ACDRCRRRKAKCEYLSAVDSCTHCRDAHVQCTFDLPLARRGPKARKKSDQPGQPPPDPSS
ace3-LC    ACDRCRRRKAKCEYLSAVDSCTHCRDAHVQCTFDLPLARRGPKARKKSDQPGQPPPDPSS
 ace3-S    PPLTNALPRPKCEYLSAVDSCTHCRDAHVQCTFDLPLARRGPKARKKSDQPGQPPPDPSS
ace3-SC    PPLTNALPRPKCEYLSAVDSCTHCRDAHVQCTFDLPLARRGPKARKKSDQPGQPPPDPSS
                Zn2Cys6 binuclear cluster domain

ace3-WT    LSTAARPGQMPPPLTFSGPAVAALQPFASSSLSPDAAWEPVEPLSIDNGLPRQPLGDLPG
ace3-EL    LSTAARPGQMPPPLTFSGPAVAALQPFASSSLSPDAAWEPVEPLSIDNGLPRQPLGDLPG
 ace3-L    LSTAARPGQMPPPLTFSGPAVAALQPFASSSLSPDAAWEPVEPLSIDNGLPRQPLGDLPG
ace3-LN    LSTAARPGQMPPPLTFSGPAVAALQPFASSSLSPDAAWEPVEPLSIDNGLPRQPLGDLPG
ace3-LC    LSTAARPGQMPPPLTFSGPAVAALQPFASSSLSPDAAWEPVEPLSIDNGLPRQPLGDLPG
 ace3-S    LSTAARPGQMPPPLTFSGPAVAALQPFASSSLSPDAAWEPVEPLSIDNGLPRQPLGDLPG
ace3-SC    LSTAARPGQMPPPLTFSGPAVAALQPFASSSLSPDAAWEPVEPLSIDNGLPRQPLGDLPG
```

Figure S2. **Protein sequence alignment of different ACE3 variants.** The Zn_2Cys_6 binuclear cluster domain is highlighted in a black box and its cysteine residues are in red. The putative nuclear localization signals (NLS), and the coiled-coil regions are in shade. The putative nuclear export signal (NES) is highlighted in a red box. The amino acids of position -17 to -7 of the C-terminus are in bold and underlined.

```
ace3-WT   LSTIQNISTRQRWIHLANAMTLRNTTLERVSKRCIDLFFDYLYPLTPLVYEPALRDVLAY
ace3-EL   LSTIQNISTRQRWIHLANAMTLRNTTLERVSKRCIDLFFDYLYPLTPLVYEPALRDVLAY
 ace3-L   LSTIQNISTRQRWIHLANAMTLRNTTLERVSKRCIDLFFDYLYPLTPLVYEPALRDVLAY
ace3-LN   LSTIQNISTRQRWIHLANAMTLRNTTLERVSKRCIDLFFDYLYPLTPLVYEPALRDVLAY
ace3-LC   LSTIQNISTRQRWIHLANAMTLRNTTLERVSKRCIDLFFDYLYPLTPLVYEPALRDVLAY
 ace3-S   LSTIQNISTRQRWIHLANAMTLRNTTLERVSKRCIDLFFDYLYPLTPLVYEPALRDVLAY
ace3-SC   LSTIQNISTRQRWIHLANAMTLRNTTLERVSKRCIDLFFDYLYPLTPLVYEPALRDVLAY

ace3-WT   IFSQPLPGVNQPSPLSQLTPDPTTGTTPLNAAESWAGFGQPSGSRTVGSRLAPWADSTFT
ace3-EL   IFSQPLPGVNQPSPLSQLTPDPTTGTTPLNAAESWAGFGQPSGSRTVGSRLAPWADSTFT
 ace3-L   IFSQPLPGVNQPSPLSQLTPDPTTGTTPLNAAESWAGFGQPSGSRTVGSRLAPWADSTFT
ace3-LN   IFSQPLPGVNQPSPLSQLTPDPTTGTTPLNAAESWAGFGQPSGSRTVGSRLAPWADSTFT
ace3-LC   IFSQPLPGVNQPSPLSQLTPDPTTGTTPLNAAESWAGFGQPSGSRTVGSRLAPWADSTFT
 ace3-S   IFSQPLPGVNQPSPLSQLTPDPTTGTTPLNAAESWAGFGQPSGSRTVGSRLAPWADSTFT
ace3-SC   IFSQPLPGVNQPSPLSQLTPDPTTGTTPLNAAESWAGFGQPSGSRTVGSRLAPWADSTFT
                                                 Coiled-coil I
ace3-WT   LVTAVCAEAAFMLPKDIFPEGESVSEILLEASRDCLHQHLEADLENPTANSIAIRYFHSN
ace3-EL   LVTAVCAEAAFMLPKDIFPEGESVSEILLEASRDCLHQHLEADLENPTANSIAIRYFHSN
 ace3-L   LVTAVCAEAAFMLPKDIFPEGESVSEILLEASRDCLHQHLEADLENPTANSIAIRYFHSN
ace3-LN   LVTAVCAEAAFMLPKDIFPEGESVSEILLEASRDCLHQHLEADLENPTANSIAIRYFHSN
ace3-LC   LVTAVCAEAAFMLPKDIFPEGESVSEILLEASRDCLHQHLEADLENPTANSIAIRYFHSN
 ace3-S   LVTAVCAEAAFMLPKDIFPEGESVSEILLEASRDCLHQHLEADLENPTANSIAIRYFHSN
ace3-SC   LVTAVCAEAAFMLPKDIFPEGESVSEILLEASRDCLHQHLEADLENPTANSIAIRYFHSN

ace3-WT   CLHAAGKPKYSWHIFGEAIRLAQVMQLHEEAALEGLVPIEAEFRRRCFWILYLGDKSAAI
ace3-EL   CLHAAGKPKYSWHIFGEAIRLAQVMQLHEEAALEGLVPIEAEFRRRCFWILYLGDKSAAI
 ace3-L   CLHAAGKPKYSWHIFGEAIRLAQVMQLHEEAALEGLVPIEAEFRRRCFWILYLGDKSAAI
ace3-LN   CLHAAGKPKYSWHIFGEAIRLAQVMQLHEEAALEGLVPIEAEFRRRCFWILYLGDKSAAI
ace3-LC   CLHAAGKPKYSWHIFGEAIRLAQVMQLHEEAALEGLVPIEAEFRRRCFWILYLGDKSAAI
 ace3-S   CLHAAGKPKYSWHIFGEAIRLAQVMQLHEEAALEGLVPIEAEFRRRCFWILYLGDKSAAI
ace3-SC   CLHAAGKPKYSWHIFGEAIRLAQVMQLHEEAALEGLVPIEAEFRRRCFWILYLGDKSAAI
```

Figure S2 continued.

```
ace3-WT   LNNRPITIHKYCFDAGITTLYPSGIEDEFLSTASEPPRKSFISGFNANVRLWQSAADLLL
ace3-EL   LNNRPITIHKYCFDAGITTLYPSGIEDEFLSTASEPPRKSFISGFNANVRLWQSAADLLL
 ace3-L   LNNRPITIHKYCFDAGITTLYPSGIEDEFLSTASEPPRKSFISGFNANVRLWQSAADLLL
ace3-LN   LNNRPITIHKYCFDAGITTLYPSGIEDEFLSTASEPPRKSFISGFNANVRLWQSAADLLL
ace3-LC   LNNRPITIHKYCFDAGITTLYPSGIEDEFLSTASEPPRKSFISGFNANVRLWQSAADLLL
 ace3-S   LNNRPITIHKYCFDAGITTLYPSGIEDEFLSTASEPPRKSFISGFNANVRLWQSAADLLL
ace3-SC   LNNRPITIHKYCFDAGITTLYPSGIEDEFLSTASEPPRKSFISGFNANVRLWQSAADLLL
           Coiled-coil II
ace3-WT   EIRVLQDQMMQHFRGTMPPNHVLPSADRQHLDSLYVRFITCLDDLPPYLQSCTLAMAAMA
ace3-EL   EIRVLQDQMMQHFRGTMPPNHVLPSADRQHLDSLYVRFITCLDDLPPYLQSCTLAMAAMA
 ace3-L   EIRVLQDQMMQHFRGTMPPNHVLPSADRQHLDSLYVRFITCLDDLPPYLQSCTLAMAAMA
ace3-LN   EIRVLQDQMMQHFRGTMPPNHVLPSADRQHLDSLYVRFITCLDDLPPYLQSCTLAMAAMA
ace3-LC   EIRVLQDQMMQHFRGTMPPNHVLPSADRQHLDSLYVRFITCLDDLPPYLQSCTLAMAAMA
 ace3-S   EIRVLQDQMMQHFRGTMPPNHVLPSADRQHLDSLYVRFITCLDDLPPYLQSCTLAMAAMA
ace3-SC   EIRVLQDQMMQHFRGTMPPNHVLPSADRQHLDSLYVRFITCLDDLPPYLQSCTLAMAAMA

ace3-WT   EGNGSAESKQYVIQCINLQVTFHCLRMVITQKFEDLSYFAPGVEQADLRKSEIVRDMLRV
ace3-EL   EGNGSAESKQYVIQCINLQVTFHCLRMVITQKFEDLSYFAPGVEQADLRKSEIVRDMLRV
 ace3-L   EGNGSAESKQYVIQCINLQVTFHCLRMVITQKFEDLSYFAPGVEQADLRKSEIVRDMLRV
ace3-LN   EGNGSAESKQYVIQCINLQVTFHCLRMVITQKFEDLSYFAPGVEQADLRKSEIVRDMLRV
ace3-LC   EGNGSAESKQYVIQCINLQVTFHCLRMVITQKFEDLSYFAPGVEQADLRKSEIVRDMLRV
 ace3-S   EGNGSAESKQYVIQCINLQVTFHCLRMVITQKFEDLSYFAPGVEQADLRKSEIVRDMLRV
ace3-SC   EGNGSAESKQYVIQCINLQVTFHCLRMVITQKFEDLSYFAPGVEQADLRKSEIVRDMLRV

ace3-WT   MNEAPFWGLQANGEPNV----------------------EKIRLIGASLLAIIHRNQDSP
ace3-EL   MNEAPFWGLQANGEPNV----------------------EKIRLIGASLLAIIHRNQDSP
 ace3-L   MNEAPFWGLQANGEPNV----------------------EKIRLIGASLLAIIHRNQDSP
ace3-LN   MNEAPFWGLQANGEPNV----------------------EKIRLIGASLLAIIHRNQDSP
ace3-LC   MNEAPFWGLQANGEPNV----------------------EKIRLIGASLLAIIHRNQDSP
 ace3-S   MNEAPFWGLQANGEPNVSRFLPRHLLLICPDYVVWMLQVEKIRLIGASLLAIIHRNQDSP
ace3-SC   MNEAPFWGLQANGEPNVSRFLPRHLLLICPDYVVWMLQVEKIRLIGASLLAIIHRNQDSP
              Putative coiled-coil
ace3-WT   LATRARSDFSVLLDILTRLDSKASDQLRNTSTTVVG*
ace3-EL   LATRARSDFSVLLDILTRLDSKASD*------------
 ace3-L   LATRARSDFSVLLDILTRLDSKASD*------------
ace3-LN   LATRARSDFSVLLDILTRLDSKASD*------------
ace3-LC   LATRARSDFSVLLDILTRLDSKASDQLRNTSTTVVG*
 ace3-S   LATRARSDFSVLLDILTRLDSKASD*------------
ace3-SC   LATRARSDFSVLLDILTRLDSKASDQLRNTSTTVVG*
                    Putative NES
```

Figure S2 continued.

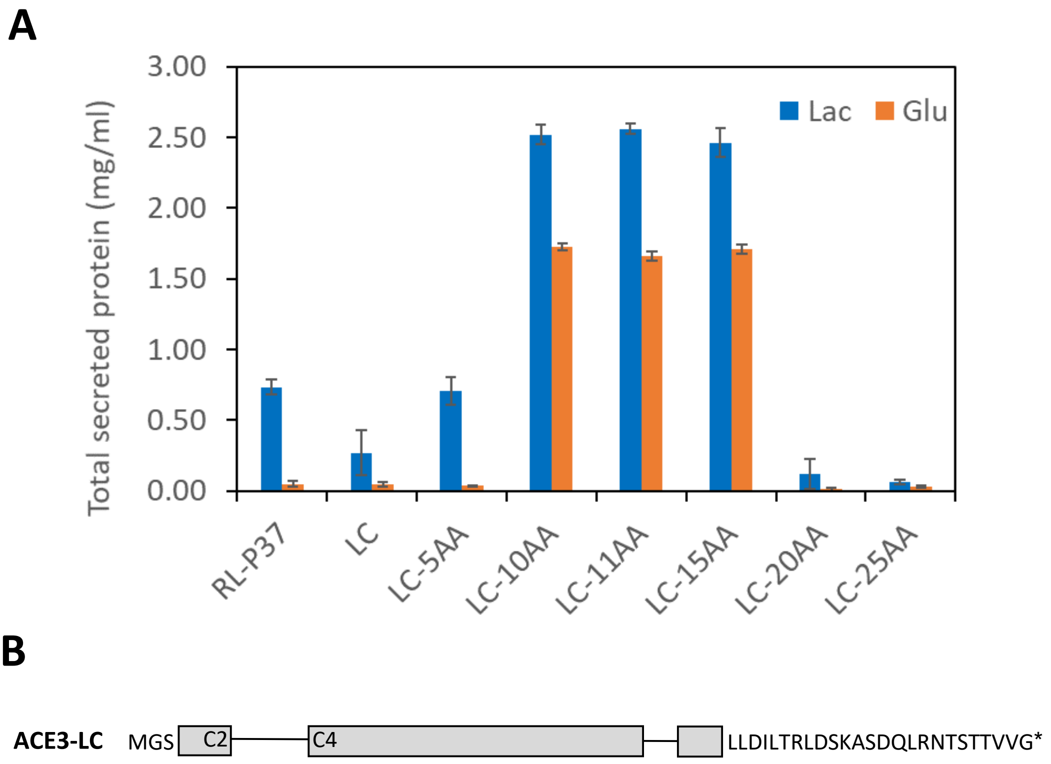

Figure S3. ACE3-LC variant with C-terminus truncation at a 5-amino acid increment. (A) Total secreted protein concentration as measured by Bradford assays. Lac, lactose; Glu, glucose. **(B)** Schematic representation of the ACE3-LC variant protein with a wild-type C-terminus. Exons are shown as boxes and introns as single lines. C2 and C4 indicate the presence of 2 and 4 cysteines of the Zn2Cys6 binuclear domain within exons, respectively. The first 3 amino acids at the N-terminus and the last 25 amino acids at the C-terminus are shown.

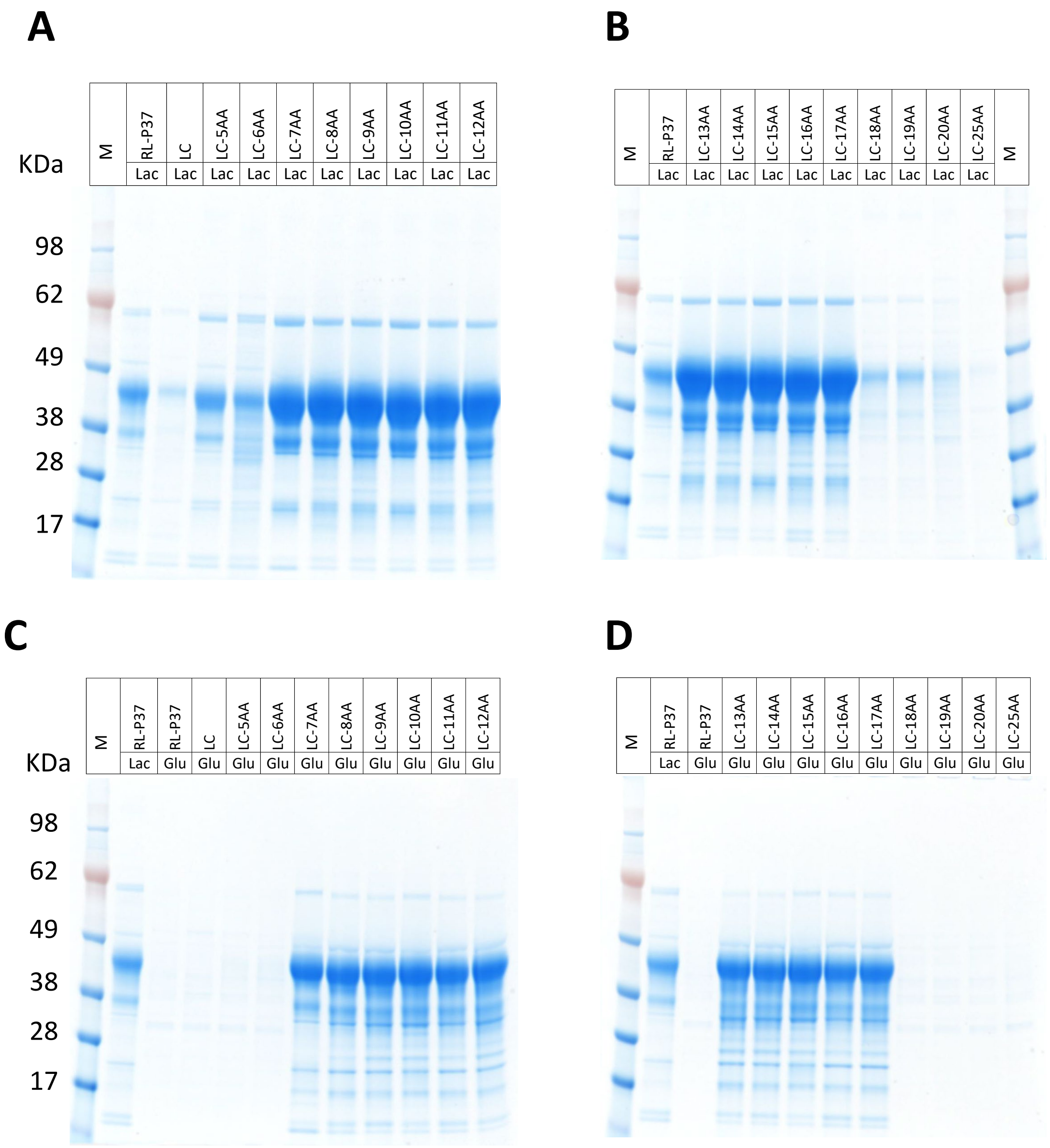

Figure S4. SDS-PAGE with strains over-expressing ACE3-LC variant with C-terminus truncation. Equal volume of culture supernatant was loaded in each lane. **(A) and (B)** are secreted protein from cells grown on lactose; **(C) and (D)** were secreted protein from cells grown on glucose. Samples from parental strain RL-P37 grown on lactose were loaded in each gel as a reference. Lac, lactose; Glu, glucose; M, protein molecular weight marker.

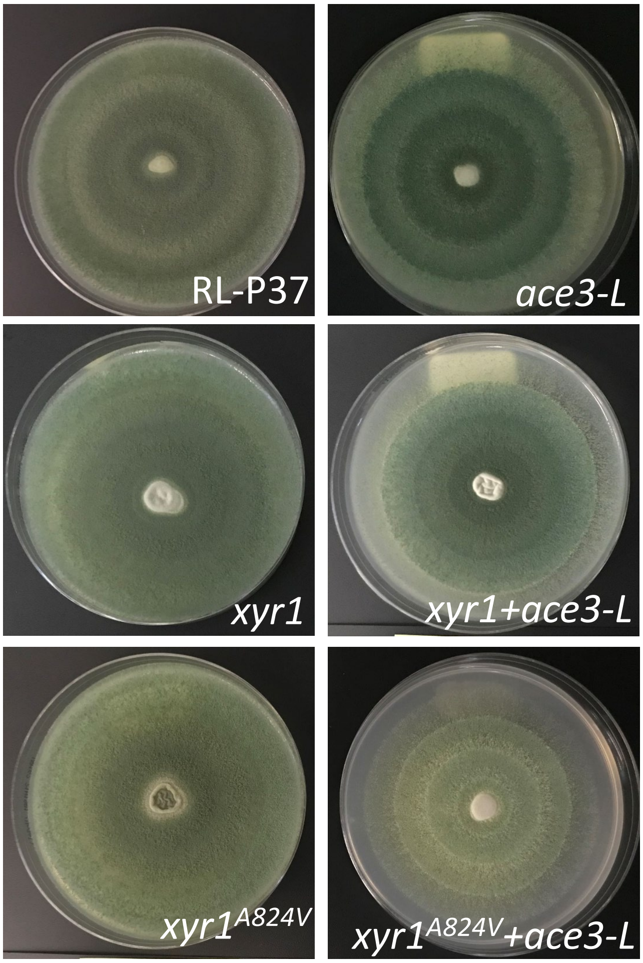

Figure S5. Growth of the parental strain (RL-P37) and its daughter strains over-expressing *xyr1* and/or *ace3* variants on Vogel's plate at 28°C for 5 days, with an alternate light/dark cycle (12hrs light:12hrs dark).

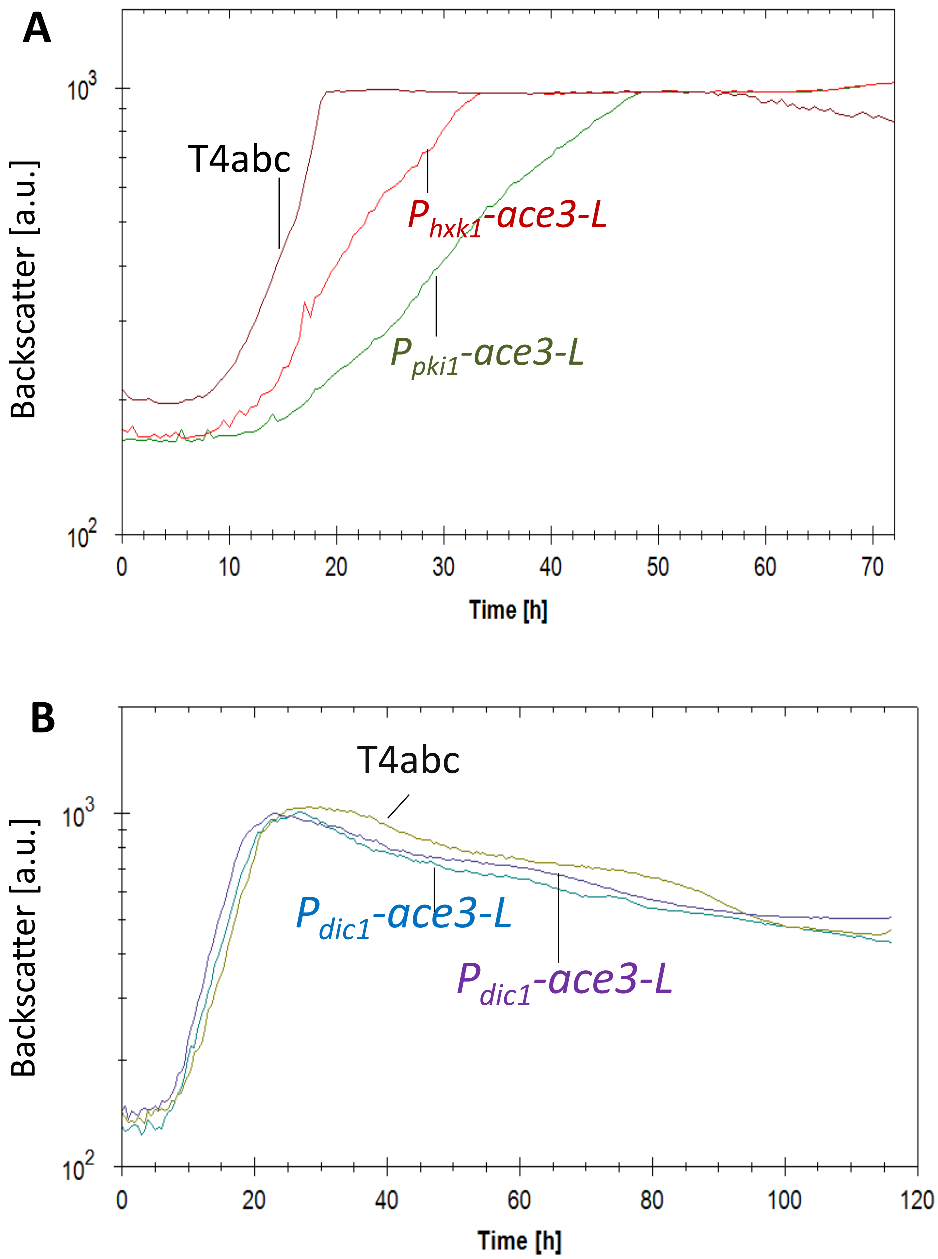

Figure S6. Growth of the parental T4abc and its daughter strains expressing the *ace3-L* variant driven by a *hxk1* promoter or a *pki1* promoter (A), and the daughter strain with *ace3-L* expression driven from a *dic1* promoter (B). Cells were grown in NREL medium supplemented with 2.5% glucose, and the growth was measured using BioLector.

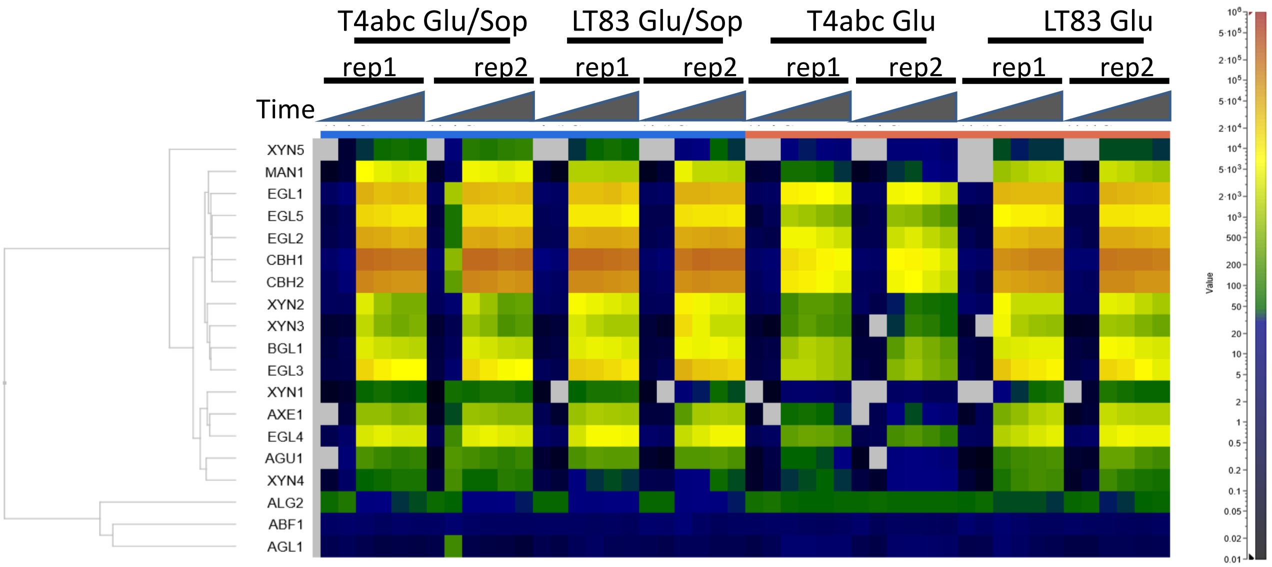

Figure S7. Heat map visualization of expression data on the genes encoding cellulases and hemicellulases in parental strain T4abc and engineered strain LT83 expressing *ace3-L*. The color code indicates the respective expression values. Expression data from 6 time points are shown in plot, including late batch growth phase, the onset of glucose/sophorose feed (0h), and 24h, 48h, 96h, and 120h after feed start. Glu, glucose; Glu/Sop, glucose/sophorose.
