# Supplementary material for: Modification of transcriptional factor ACE3 enhances protein production in Trichoderma reesei in the absence of cellulase gene inducer
Source: Biotechnol Biofuels. 2020 Aug 6;13:137. doi: 10.1186/s13068-020-01778-w (PMC7412840; doi:10.1186/s13068-020-01778-w)
Supplement: Supplementary file 3 — Additional file 3: Supplemental materials and methods. The materials and methods include RNA-sequencing and data analysis, protein sequence alignment and motif search, and growth curve with BioLector. [file 13068_2020_1778_MOESM3_ESM.docx]

**Supplemental Material and Methods**

**RNA-sequencing and data analysis**

**QM9414 RNAseq analysis at *ace3* locus**

The QM9414 RNA sequencing datasets were obtained from NCBI sequence read archive accession SRP034709, consisting of *T. reesei* QM9414 grown on different cellulase inducing growth media: 2% glucose, 1% cellulose, and 2% glucose with 1 mM sophorose [1]. Data corresponding to 24 hours post cellulase induction were mapped to the *T. reesei* QM6a genome [2] (using Hisat2 version 2.1.0 [3]), alignments were normalized using deepTools bamCoverage (--normalizeUsing BPM) version 3.3.1 [4], and subsequently visualized with pyGenomeTracks version 3.1.2 [5].

**RNAseq analysis with propriety strains and heatmap of cellulase and hemicellulase genes.**

Parental strain (T4abc) and its daughter strain expressing the *ace3-L* variant (LT83) were fermented as described above and mycelia samples were periodically collected, washed with sterile water and immediately froze in liquid nitrogen. Cells were mechanically lysed with bead beating and total RNA was isolated using Qiagen RNeasy (Qiagen, USA. Catalog No. 74106). Next generation sequencing was completed using TruSeq Stranded mRNA Library Prep Kit (Illumina, USA; Part # RS-122-2101), sequenced on an Illumina HiSeq 2500 system (single ended reads for 75 cycles). RNA sequencing data was quantified using Kallisto version 0.46.1 [6] against the *T. reesei* transcriptome. A heatmap of gene expression of select cellulase and hemicellulases was generated using Genedata Analyst v9.5.

**Protein sequence alignment and motif searches.**

Multiple alignments of ACE3 variant protein sequences were performed with ClustalW2 (http://www.ebi.ac.uk/Tools/msa/clustalw2/). Predicted Nuclear localization signals (NLS) in the amino acid sequences were determined using the NucPred (https://nucpred.bioinfo.se/nucpred/) [7] and predicted nuclear export signals (NES) were detected with the NetNES Server (<http://www.cbs.dtu.dk/services/NetNES/>) [8].

**Growth curves with BioLector.**

For growth experiments, cells were precultured in a 24-deep-well plate with 2 ml YEG broth for 48 hours at 32°C with shaking. One hundred microliter of the preculture were inoculated in 1.25 ml of NREL medium supplemented with 2.5% (w/v) glucose in a 48-well FlowerPlate (m2p-labs, Baesweiler, Germany), cultivated in a BioLector microcultivation system (m2p-labs, Baesweiler, Germany) at 800 rpm, 30°C and 80% humidity. Growth was followed by measuring the backscattered light, which reflects the cell density [9, 10].

## **Reference**

1. Dos Santos Castro, L., et al., *Comparative metabolism of cellulose, sophorose and glucose in Trichoderma reesei using high-throughput genomic and proteomic analyses.* Biotechnol Biofuels, 2014. **7**(1): p. 41.

2. Li, W.C., et al., *Trichoderma reesei complete genome sequence, repeat-induced point mutation, and partitioning of CAZyme gene clusters.* Biotechnol Biofuels, 2017. **10**: p. 170.

3. Kim, D., B. Langmead, and S.L. Salzberg, *HISAT: a fast spliced aligner with low memory requirements.* Nat Methods, 2015. **12**(4): p. 357-60.

4. Ramirez, F., et al., *deepTools2: a next generation web server for deep-sequencing data analysis.* Nucleic Acids Res, 2016. **44**(W1): p. W160-5.

5. Ramirez, F., et al., *High-resolution TADs reveal DNA sequences underlying genome organization in flies.* Nat Commun, 2018. **9**(1): p. 189.

6. Bray, N.L., et al., *Near-optimal probabilistic RNA-seq quantification.* Nat Biotechnol, 2016. **34**(5): p. 525-7.

7. Brameier, M., A. Krings, and R.M. MacCallum, *NucPred--predicting nuclear localization of proteins.* Bioinformatics, 2007. **23**(9): p. 1159-60.

8. la Cour, T., et al., *Analysis and prediction of leucine-rich nuclear export signals.* Protein Eng Des Sel, 2004. **17**(6): p. 527-36.

9. Kensy, F., et al., *Validation of a high-throughput fermentation system based on online monitoring of biomass and fluorescence in continuously shaken microtiter plates.* Microb Cell Fact, 2009. **8**: p. 31.

10. Jansen, R.P., et al., *A closer look at Aspergillus: online monitoring via scattered light enables reproducible phenotyping.* Fungal Biol Biotechnol, 2019. **6**: p. 11.
